# Projecting nitrous oxide over the 21st century, uncertainty related to stratospheric loss

**DOI:** 10.1073/pnas.2524123123

**Published:** 2026-02-02

**Authors:** Michael J. Prather, Calum P. Wilson

**Affiliations:** ^a^Earth System Science Department, University of California, Irvine, CA 92697-3100

**Keywords:** nitrous oxide, lifetimes, climate change, ozone depletion, greenhouse gases

## Abstract

Extending the N_2_O lifetime derived from Microwave Limb Sounder satellite observations, we find a mean value of 117 y and a likely decrease of –1.4 ± 0.9% per decade over the period 2004 to 2024. This trend is consistent with the previously published 2004 to 2021 value of –2.1 ± 1.2% per decade. A more careful analysis of uncertainty now provides a more robust likely (one-sigma) range. From analyses of a range of factors controlling the N_2_O lifetime, we find that the decrease in lifetime can be explained by recent changes in stratospheric circulation and temperature. Projection of the lifetime change to 2100 shows that this effect is comparable to differences across the shared socioeconomic pathways used for climate projections and cannot be ignored. An updated evaluation of the N_2_O chemical feedbacks shows that this effect produces a relatively small shift in atmospheric abundance over the 21st century, but still an important shift, –11%, in the global warming potential of N_2_O.

## Significance

Projecting atmospheric nitrous oxide (N_2_ O) abundance is critical for climate and ozone assessments. Research has focused on projecting the changing emissions of N_2_ O from direct anthropogenic sources, the dominant cause of the recent growth. Earth system models are now projecting natural sources perturbed by climate change. There has been little effort to understand how climate and compositional changes may change the stratospheric sink of N_2_ O, which balances all these sources and also controls the atmospheric abundance. Here, we review recent observational and modeling evidence for an increase in the sink caused by decreasing N_2_ O lifetime and show that it introduces uncertainties comparable to shifts across the different shared socioeconomic pathway scenarios used in current assessments.

Nitrous oxide (N_2_O) is a major greenhouse gas (1, 2) and ozone-depleting gas (3, 4). Its tropospheric abundance—about 337 ppb (mol Gmol^−1^ of dry air) in 2024 (5)—is increasing steadily at about 3% dec^−1^ due to human interference with the natural cycle of nitrogen fixation. The growth rate shows that N_2_O is greatly out of balance (6) with sources exceeding sinks by about 35%, and the imbalance is attributed to human activities (7). The primary sink of N_2_O is from photochemical destruction in the stratosphere, and recent analyses give a lifetime (i.e., total atmospheric burden divided by stratospheric loss) of about 117 y (8, 9). Most current efforts to understand N_2_O trends have focused on sources, derived from either bottom–up sums over individual emission types (7) or top–down atmospheric inversions (10). This attention is logical since the rise in N_2_O over the industrial era, including its current growth rate, is driven predominantly by increased sources. Here, we focus on N_2_O sinks and assess their uncertainties and trends, calculating the impact on N_2_O projections over this coming century.

## Stratospheric N_2_O-NOy Chemistry

N_2_O accumulates in the lower atmosphere until it is transported into the tropical stratosphere by the Brewer–Dobson Circulation (BDC) (11, 12), where photochemistry destroys it. Table 1 summarizes the key rates involving N_2_O and NOy (the odd-nitrogen family comprising NO, NO_2_, N, and HNO_3_). The middle and upper stratosphere is where N_2_O is destroyed (R1, R5, R6) and stratospheric NO is produced (R6) (8). Most N_2_O loss (90%) is through photolysis with the remaining (10%) from reaction with excited state of atomic oxygen, O(^1^D) (13–15). The levels of O(^1^D) are set by a balance between O_3_ photolysis (R2) and collisional quenching by N_2_ and O_2_ (R3, R4).

**Table 1.** Stratospheric chemical rates controlling N_2_O and NOy

Rate	Reaction		Products	Rate coefficient	Fraction of total loss (strat, trop)
R1	N_2_O + *hv*		N_2_ + O(^1^D)	*dlnR/dT:* +0.4% K^−1^	(90%, 0%)
R2	O_3_ + *hv*		O(^1^D) + O_2_	*dlnR/dT:* +0.2% K^-1^	
R3	O(^1^D) + N_2_	→	O + N_2_	2.15 × 10^−11^ e^(+110/T)^	
R4	O(^1^D) + O_2_	→	O + O_2_	3.30 × 10^−11^ e^(+55/T)^	
R5	N_2_O + O(^1^D)	→	N_2_ + O_2_	4.64 × 10^−11^ e^(+20/T)^	(4.5%, 0.5%)
R6	N_2_O + O(^1^D)	→	NO + NO	7.26 × 10^−11^ e^(+20/T)^	(5.5%, 0.5%)
R7	NO + *hv*	→	N + O		
R8	N + O_2_	→	NO + O	3.30 × 10^−12^ e^(−3150/T)^	
R9	N + NO	→	N_2_ + O	2.10 × 10^−11^ e^(+100/T)^	
R10	N + NO_2_	→	N_2_O + O	5.80 × 10^−12^ e^(+220/T)^	(–1%, 0%)
R11	NO + O_3_	→	NO_2_ + O_2_		
R12	NO_2_ + O	→	NO + O_2_	5.30 × 10^−12^ e^(+200/T)^	

Notes: In the middle and upper tropical stratosphere, where most of the N_2_O loss and NO production occurs, the odd-nitrogen family NOy is predominantly N, NO, NO_2_, and HNO_3_. Sensitivity of photolysis rates (R1, R4) to temperature (*dlnR/dT*) is based on the temperature dependence of the cross sections and calculated for 24 to 34 km range in the tropics. Rate coefficients are taken from ref. 16. Loss fractions are primarily stratospheric (100%) plus 1% from R5 & R6 in the troposphere and 1% production from R10 (13–15).

The NO produced in one of the three N_2_O loss channels (R6) is the primary source of stratospheric NOy, which drives catalytic destruction of O_3_ (R11, R12), and is the reason why N_2_O is an ozone depleting substance. Most of the NOy is transported into the lowermost stratosphere outside the tropics via the BDC and thence into the troposphere. About a third of the NOy produced is destroyed in the stratosphere and mesosphere through photolysis of NO (R7) followed by reaction of atomic N with NO (R9). This NOy model is consistent with the observed NOy–N_2_O tracer slope in the lowermost stratosphere (13). The balance between these two NOy sinks, and thus the amount of NOy in the stratosphere, is very temperature sensitive. More than 90% of the atomic N produced is recycled back to NO (R8), but this rate coefficient is very sensitive to temperature (+4.3% K^−1^). Rosenfeld and Douglass (17) noted that with a CO_2_-driven cooling of the stratosphere over the 21st century, R8 decreases by tens of percent while R9 hardly changes. Thus, overall NOy:N_2_O ratio declines by similar percentages and less O_3_ is destroyed. In addition, part of the NOy catalytic cycle is temperature sensitive (R11, +2.1% K^−1^), and CO_2_ cooling will reduce O_3_ destruction even at constant NOy levels.

## N_2_O Lifetime

The atmospheric chemistry community has been remarkably vague about the formal definition of lifetime for gases like N_2_O. Overall, it is accepted that the lifetime is defined as the overall burden (atmospheric content in kg) divided by the sinks (kg per year), see various definitions in ref. 18. First, the lifetime of any gas, including N_2_O, depends on where and when it is emitted (19). For example, the N_2_O produced in the stratosphere (R10) has a much shorter lifetime than that emitted at the surface (<10 y vs. >100 y). Most (99%) of N_2_O sources are at the surface and become well mixed in the troposphere before being transported into the stratosphere where N_2_O is photochemically destroyed (see reactions Table 1). Thus, we calculate an N_2_O lifetime for surface emissions as 117 y (9).

**Fig. 1.**
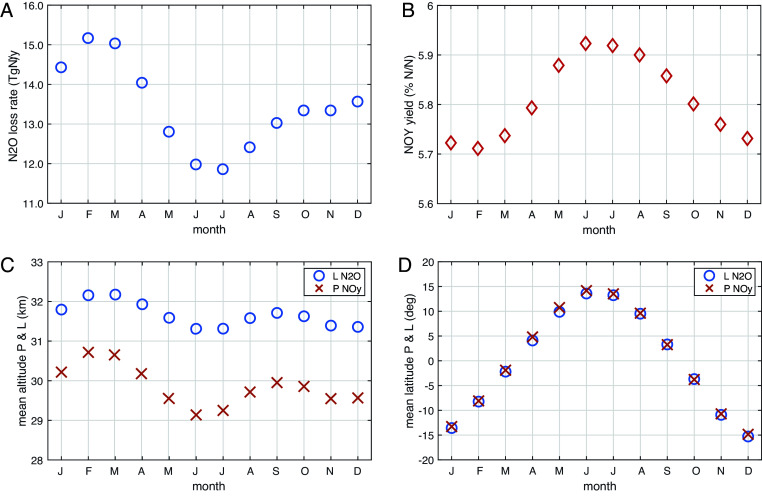
Observation-model-derived budgets for N_2_O and NOy. (*A*) Monthly annual cycle of N_2_O loss rate (TgN/y) calculated from the MLS monthly data for N_2_O, O_3_, and T for Jan 2005 through Dec 2024. (*B*) Annual cycle of NOy yield (% per N_2_O lost as N per N). (*C*) Mean altitude (km, in pressure altitude) of the N_2_O loss (“o”) and NOy production (“x”). (*D*) Mean latitude (degrees) of the N_2_O loss (o) and NOy production (x).

Additional ambiguity arises when we calculate the total atmospheric loss rate (e.g., kg-N lost per year) from a set of monthly mean values, i.e., how to integrate these losses over a year, considering leap years. The standard year is 365 d of 86,400 s; the Julian year, 365 d 6 h, approximates our current leap-year calendar; and the true sidereal year is slightly longer, 365 d 6 h 9 m 10 s. For a temporally uniform loss rate, the leap years will introduce a false interannual variation of 0.3 y in a 117 y lifetime. February is also the peak month of N_2_O loss (Fig. 1*A*). Hence, we assume all February’s are weighed equally (28.25 d) and retain the Julian year.

## Current N_2_O Variability and Trends from MLS Observations

Using the methodology of ref. 8 to calculate N_2_O loss, ref. 9 examined an extended time series (2004 to 2021) of MLS observations of O_3_, T, and N_2_O (20–22) and found that the N_2_O lifetime was declining at −2.1% dec^−1^ because N_2_O abundances were increasing in the primary destruction region of the middle and upper tropical stratosphere faster than in the troposphere. With three additional years for a longer record (2004 to 2024), we look for further changes in trends and variability.

**Fig. 2.**
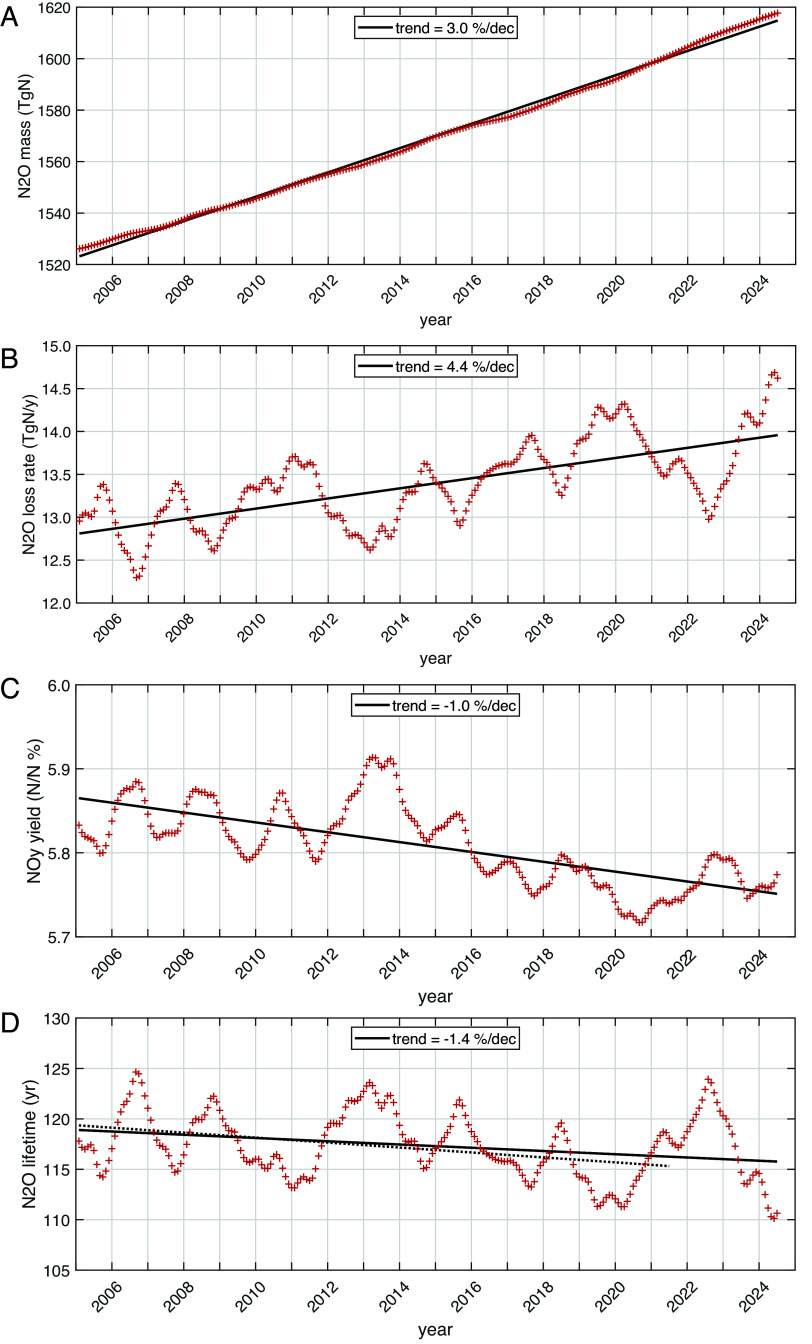
IAV of key N_2_O quantities. All data here have the annual cycle removed (Fig. 1) by summing or averaging over 1 y with daily weighting for each month from Aug 2004 through Dec 2024. Every Feb has 28.25 d. The first point covers Aug 2004 through Jul 2005 and is plotted as 1 Feb 2005. The last point is the sum over Jan 2024 through Dec 2024 and plotted as 1 Jul 2024. (*A*) N_2_O total atmospheric mass (TgN) derived from NOAA monthly global mean surface observations (ref) and a scaling factor of 4.79 TgN/ppb. The linear trend line fit (+3.0%/dec) shows the positive residuals of the fit at both ends, indicating the parabolic pattern of the N_2_O growth rate, corresponding to a near uniform acceleration of 2.1% y^−2^. (*B*) N_2_O loss rate (TgN/y) calculated from the MLS monthly data for N_2_O, O_3_, and T for Aug 2004 through Dec 2024. The trend line (+4.4%/dec) shows that N_2_O loss is increasing faster than the burden. The IAV is much smaller than the annual cycle (Fig. 1*A*) and correlates with QBO winds (Fig. 3*A*). (*C*) Yield rate of NO (%) in terms of moles of N produced per moles of N lost of N_2_O. (*D*) Annual mean lifetime of N_2_O (y) derived from panels (*A*) and (*B*). The solid black trend line (–1.4%/dec) for the extended period is compared with that from previous analysis using data through Dec 2021 (dotted line, –2.1%/dec, 9). The mean lifetime for either period is 117.3 y.

The annual cycle of the N_2_O-NOy chemistry continues to show stable annual patterns that provide a useful test for chemistry-climate models (as in figure 4 of ref. 8). We update the annual cycles in Fig. 1. The monthly mean loss rate, L-N_2_O, peaks at 15.2 TgN y^−1^ in Feb–Mar with a June to July minimum of 11.9 TgN y^−1^ (Fig. 1*A*). The sinusoidal max–min seasonal amplitude (defined as 2^3/2^ × SD) is 3.1 TgN y^−1^, which is much larger than the quasi-biennial oscillation (QBO, 24 to 28 mo) interannual variability (IAV) amplitude of 1.0 TgN y^−1^ (Fig. 2*B*). This annual cycle cannot be explained by the sun–earth distance but must be due to an annual cycle in tropical upwelling that peaks in Feb just after perihelion, in addition to the QBO IAV signal (figure 3 of ref. 8). The yield of NOy from N_2_O loss averages about 5.8% (N per N) with a very small annual cycle and a Jun–Jul peak in opposite phase to the N_2_O loss (Fig. 1*B*). This phase difference is due to the altitude separation of L-N_2_O (centered on ~32 km) from NOy production (P-NOy, ~30 km), see Fig. 1*C* and also ref. 8. Note that NOy yield has very small annual amplitude (<2%) compared to L-N_2_O (13%), and thus a plot of P-NOy would look mostly like L-N_2_O with a slightly smaller amplitude. The mean latitude of L-N_2_O and P-NOy (Fig. 1*D*) coincide and follow the annual solar declination cycle, but with smaller range, ±15° vs. ±23.5°.

From the extended monthly record of tropospheric surface N_2_O observations from NOAA (5), we create a 1-y, day-weighted, IAV record of global N_2_O burden (TgN, see Fig. 2*A*), which has a trend of +3.0% dec^−1^. The linear fit (solid black line) clearly shows the positive residuals of the fit at both ends, indicating the parabolic pattern of the N_2_O growth and corresponding to a near uniform acceleration of 2.1% y^−2^ over the 19 y record shown here. As with the burden, the annual total L-N_2_O (TgN y^−1^, Fig. 1*B*) is now calculated with more care for the impact of leap years. With the Julian year, each February’s loss rate (e.g., in TgN d^−1^) uses 28.25 d and thus has the same weighting, whether a leap year or not. The L-N_2_O growth rate is slightly lower than in ref. 9 (+4.4 vs. +5.0% dec^−1^) but is still distinctly higher than the burden increase. The NOy production increases in parallel with L-N_2_O, but at a slower rate. We show the NOy yield (Fig. 2*C*) with its clear, but small decline, −1.0% dec^−1^, in order to highlight the separation of the two distinct chemical mechanisms: L-N_2_O increases occur mostly in the upper stratosphere where the NOy yield is much smaller. The lifetime, derived from Fig. 2 *A* and *B*, was found in ref. 9 to be decreasing at a rate of –2.1% dec^−1^ (dotted line in Fig. 1*D*), and with the addition of 3 more years of MLS data, the decrease is smaller, –1.4% dec^−1^. The trend in J-N_2_O (R1) from ref. 9’s Fig. 1*E* was –1% dec^−1^, which is contrary to the increasing loss rate and not reexamined here.

How robust is this negative trend in N_2_O lifetime? If we simply ask for the 1−σ CI (~68%) in a linear fit to the 234 monthly points derived from 12-mo means (1 Feb 2025 through 1 Jul 2024) and assume 232 degrees of freedom (DOFs), then the negative trend is quite robust, –1.37 ± 0.31% dec^−1^. This uncertainty range, however, is inconsistent with the trend changing from –2.1 to –1.4% dec^−1^ with the addition of 3 y of more recent data (Fig. 2*D*). It is obvious that the monthly points correlate on a quasi-biennial and shorter time scales. If we count the number of minima or maxima including the level periods, we estimate about 21 DOF. The reduction in DOF from 232 to 21 increases the SE of the slope by a factor of 3.37 (3.32 for the square root of DOF and the rest for the small shift in the t-statistic) to give an updated lifetime trend of –1.37 ± 1.04% dec^−1^. This change makes the 2004 to 2021 and 2004 to 2024 trends statistically consistent but still argues for the trend being negative.

**Fig. 3.**
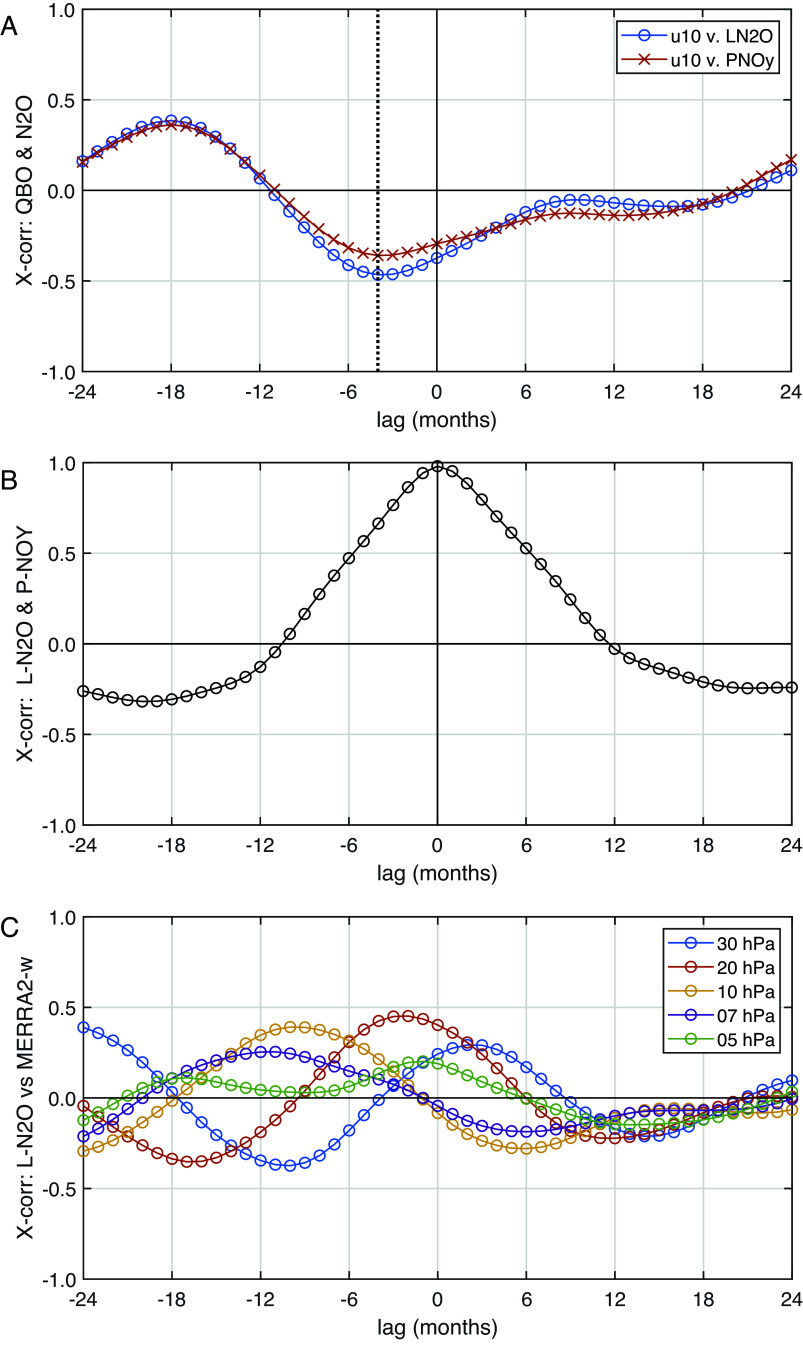
Cross correlation of N_2_O and NOy chemical rates and QBO indices. (*A*) Detrended N_2_O loss (o) and NOy production (x) against QBO metric (u_10_ = equatorial wind at 10 hPa), showing peak correlation with N_2_O-loss of –0.47 at –4 mo (vertical dotted line, N_2_O lagging QBO). All data have been yearly averaged, month-by-month. (*B*) NOy production against N_2_O loss, showing peak correlation of 0.98 and no significant lag. (*C*) N_2_O loss against residual vertical velocity w*_QBO_ at pressure levels: 30, 20, 10, 7, 5 hPa.

If we can reduce the residuals in the fit by attributing them to observed atmospheric phenomena, then we can reduce the uncertainty in the long-term trends. The obvious residual pattern looks QBO-like, and we seek a QBO index that reflects the fluctuations in N_2_O loss of which 81% occurs between 3.2 and 32 hPa as seen in refs. 8 and 9. We first try the classic measure of the QBO, the monthly mean zonal winds at 10 hPa (u_10_) from the Singapore radiosonde (23). Cross-correlation of u_10_ with N_2_O loss shows a peak Pearson’s R value of –0.47 at –4 mo (Fig. 3*A*, R^2^ = 0.22). Our N_2_O IAV chemical budgets terms (12-mo means) are generally coherent over the tropical middle stratosphere. For example, even though L-N_2_O and P-NOy are clearly separated in altitude (Fig. 1*C*), their cross-correlation has zero lag and R^2^ = 0.96 (Fig. 3*B*). Including the lagged u_10_ data in the linear fit reduces the SE by only 10%. Searching for a better QBO index, we tested u_30_ (at 30 hPa) and found a different lag (–10 mo), but similarly modest reduction in the SE. Because L-N_2_O is driven by upward transport, we tested its correlation with the monthly mean tropical residual vertical velocity w*_QBO_ derived from MERRA-2 reanalysis (24) using pressure levels encompassing the dominant N_2_O loss: 30, 20, 10, 7, 5 hPa. None of these w*_QBO_ produced a much better cross correlation (Fig. 3*C*) or reduced the standard error in the trend. This attempt failed because the phase of w*_QBO_ changes with altitude across the range of N_2_O loss. See the excellent analysis of the observed height-dependent QBO phasing of N_2_O–O_3_–NOx–HNO_3_ by Park et al. (25), who also demonstrate the ability of a chemistry-transport model to match these observations.

Our best estimate of the lifetime trend, –1.37 ± 0.92% dec^−1^ with a 68% (“likely”) CI, produces a robust trend; but, if a 90% (“very likely”) confidence level is required, the interval, –1.37 ± 1.54% dec^−1^, now includes no trend.

## Future N_2_O Trends from Climate Change

### Temperature Trends.

Atmospheric CO_2_ increases enable more efficient cooling of the stratosphere and mesosphere. Analysis of stratospheric microwave temperatures (26) indicates an overall cooling over the satellite record (1986 to 2002) of –2 °C. This cooling has been nearly uniform over the three stratospheric sounding unit (SSU) channels centered at 30, 38, and 45 km. This range covers the main (10th to 90th%ile) N_2_O loss region of 24 to 42 km (figure 1 of ref. 8). Scaling this total cooling to the period 2020 to 2100 gives –5 °C, admittedly a simple projection, since it assumes that CO_2_ and the cooling continue to grow linearly over this century. Our assessment here is primarily first-order in estimating how the cooling stratosphere will alter some key rates and species, although we do carry on to second-order effects when we recalculate the MLS L-N_2_O with an altered O_3_ column.

#### N_2_O–NO Kinetics.

The N_2_O kinetics (Table 1) have distinct but small temperature dependencies for a change of −5 °C: Cross-sections for photolysis of N_2_O (R1) drop by –2.1%; cross-sections for O_3_ yielding O(^1^D) (R2), by about –0.7%; the quenching rate coefficient for O(^1^D) (R3, R4) increases by 0.7%; and the rate coefficients for loss of N_2_O to O(^1^D) (R5, R6) increase by <0.2%. Thus, from photochemical rate coefficients alone, we expect N_2_O lifetime to increase by about 1 to 2% over the remaining 21st century. We integrated these kinetic effects by recalculating N_2_O loss over the 245 mo of MLS data (Aug 2004 to Dec 2024) after shifting all temperatures by −5 °C. The reduction in N_2_O loss (R4+R5+R6), –1.2 ± 0.0 (SD)%, was stable and consistent with the kinetics estimates above. The reduction in NO production (R5) was less, –0.9 ± 0.0%, because the largest kinetics shift was in the cross-sections (R4). Thus, on the% per decade level observed for the recent MLS-derived lifetime, these direct kinetic changes in the N_2_O–NO system fall below 0.2% dec^−1^.

#### O_3_ kinetics.

A cooling stratosphere reduces the rate of catalytic O_3_ loss and leads to increased O_3_ levels. Here, we estimated this effect by calculating the photochemical steady-state O_3_ levels (ssO_3_) for a typical tropical atmosphere with the Pratmo box-model code used for Linoz chemistry (27, 28). The ssO_3_ values for the standard simulation were within 20% of the O_3_ climatology values over the altitude range of 20 to 50 km, giving us confidence that the temperature sensitivity of ssO_3_ should reflect that of atmospheric O_3_. When the fixed climatology for the O_3_ column is retained with a shift of −5 °C, ssO_3_ increases by +7±1% over 24 to 40 km. When scaled, our steady-state results are remarkably similar to results from the 2D chemistry-transport model of ref. 17, who found a 14% stratospheric O_3_ increase for a cooling of −10 °C.

#### O_3_ changes.

To assess the impact of these O_3_ changes, both local and to the column, we recalculated the 245 mo of MLS data after increasing the MLS O_3_ values by 7%. The impact of O_3_ changes on N_2_O budgets was much larger than the T-kinetics effects. The O_3_ increase leads to a reduction in N_2_O loss (R1+R5+R6) of −5.9 ± 0.3% (increase in lifetime) that was similar across all months. The reduction in NO production (R5) is less, −1.5%, because the increased local O_3_ offsets the reduced O(^1^D) J-values (R2). On decadal scales, combining kinetics effects plus O_3_ responses to T, we now find changes in the N_2_O lifetime of order 1% dec^−1^, similar to the currently observed lifetime trends but of opposite sign.

Our ssO_3_ calculation above did not account for the change in column O_3_ as the ref. 17 model would have. Increased column O_3_ shields levels below from UV radiation, reducing photolysis of O_2_ and steady-state O_3_ levels (29). In tests with augmented O_3_ columns, we found that this column feedback had small impact above 32 km; but by 24 km it had canceled most of the T-driven increase. With peak N_2_O loss occurring at 32 km (Fig. 1*C*; figure 1 of ref. 8), this column feedback will only partly offset the anticipated decrease in N_2_O loss as the stratosphere cools.

#### Chemical feedbacks.

Estimates here are based on first- and second-order changes where the sensitivity factors driving each change are clear. The N_2_O feedback on its own lifetime is a far more complex loop connecting N_2_O–NO–O_3_–J–N_2_O–N_2_O that couples kinetics to transport to radiation (19). We cannot do this calculation without a set of carefully designed experiments using a chemistry-climate model. Because the lifetime feedback is <10%, we believe its impact on the first-order effects derived here will be proportionally smaller. Nevertheless, a key direct photochemical impact of stratospheric cooling is the reduced rate coefficient for R8 with more NO being destroyed via R9. Thus, NOy levels and O_3_ loss are reduced, and this is expected to make the lifetime feedback less important. As first noted in ref. 17, the impact of N_2_O as an ozone depleting gas is significantly reduced by the end of the century in a cooling stratosphere.

### Circulation Trends.

As noted previously (30), the negative trend in N_2_O lifetime is caused by increasing N_2_O abundances in the tropical mid-stratosphere that are relatively larger than the tropospheric burden due to more rapid upward transport. This upward transport driving N_2_O loss is a part of the stratospheric BDC overturning. From both theory and modeling, it is agreed that increasing greenhouse gases (i.e., tropospheric warming with stratospheric cooling) should enhance the BDC, and such is found in model intercomparison projects (31, 32) as well as reanalyzed meteorological data (33). Unfortunately, most of these BDC studies diagnose mass fluxes across the 100 or 70 hPa surfaces (16 or 19 km) to detect trends, and information on the tropical middle stratosphere climate-mean w* is limited. From the recent CMIP6 model results, Abalos et al. (32) found an overall increase in w* ranging from +1.5% dec^−1^ at 19 km to +1.8% dec^−1^ at 45 km. These rates are consistent with the 19 km results from ERA5 meteorology (33) but inconsistent with tracer observations (CO_2_, SF_6_) in the northern mid-latitude stratosphere (34). This mid-latitude tracer response to BDC changes depends not simply on tropical w* as N_2_O does, but on latitudinal transport out of the tropics (35). The N_2_O observations (9, 30, 36) clearly support the increase in w* and the overall BDC.

The impact of a faster tropical w* on N_2_O lifetime is partly buffered because loss occurring below 30 km where abundances fall off slowly with altitude is hardly affected by w* changes. Thus, we build a one-dimensional tropical pipe model with equatorial photolysis rates (J-N_2_O) taken from our MLS modeling: 37 model levels with regular pressure altitude levels from 0 to 48 km; N_2_O lower boundary condition of 340 ppb in the lowest layer; decay N_2_O for 90 d at each level; push each level up one (~15 m/d); and continue until a steady state is reached. Our pipe model N_2_O profile calculates a realistic shape (i.e., N_2_O half-value at ~33 km) and lifetime (column value of 101.3 y). When we increase w* by 10%, the lifetime drops by 5.5%, i.e., the sensitivity dln(N_2_O lifetime)/dln(w*) = –0.55. Thus, when tropical upwelling rates increase by +1.8% dec^−1^, the lifetime would decrease by –1.0% dec^−1^, consistent with the derived trend here, –1.37 ± 0.92% dec^−1^. This model uses fixed w* and J-N_2_O and thus does not include the N_2_O lifetime feedbacks, which we estimate to be a secondary effect.

## Projecting N_2_O

Recent projections of N_2_O for the Intergovernmental Panel on Climate Change (IPCC) (37, 38) have approximated the chemical feedbacks and circulation changes in the tropical stratosphere in various ways, but mostly as simple parameterizations based on old publications. Here, we take an updated look at how the chemical feedbacks and observed trends might affect the projections of N_2_O over the 21st century.

**Fig. 4.**
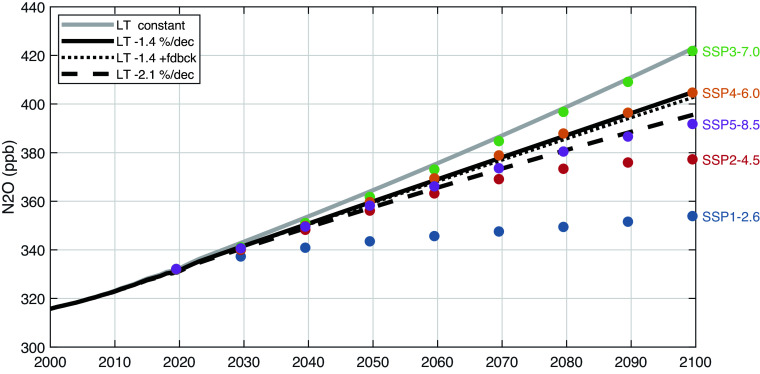
Observed (2000 to 2024) and projected (2025 to 2100) N_2_O annual mean tropospheric abundance (gray line) assuming the observed 2005 to 2024 +3.0%/decade growth rate continues to 2100. N_2_O emissions (2025 to 2100) are calculated from this projection assuming a fixed lifetime of 120 y. Using these emissions, the N_2_O abundance is projected (black lines) using three different lifetime assumptions: (solid line) lifetime decreases at –1.4% per decade; (thin dotted line) same lifetime trend plus a chemical feedback factor that reduces the lifetime with increasing burden (i.e., dln(N_2_O lifetime)/dln(N_2_O burden) = –0.065; 15); and (dashed line) the lifetime decreases at –2.1% per decade as found from the earlier period 2004 to 2021 (9). The reduction in 2100 N_2_O abundance for these three cases are –4.3, –4.5, and –6.5%, respectively. A range of SSP emissions scenario projections (SSP1-2.6, SSP2-4.5, SSP5-8.5, SSP4-6.0, SSP3-7.0, in ascending order) calculated by Meinshausen et al. (37) for the IPCC assessments are shown for perspective.

For our baseline N_2_O scenario (thick gray solid line in Fig. 4), we use the NOAA tropospheric-mean abundance up to 2024 and then project it for the rest of the 21st century at +3.0% dec^−1^ (recognizing that this is likely an underestimate based on the curvature in recent years). Comparing our baseline with a sample of IPCC shared socio-economic pathway (SSP) scenarios (SSP1-2.6, SSP2-4.5, SSP5-8.5, SSP4-6.0, SSP3-7.0, plotted as decadal colored dots in ascending order (37, 39), we see that our baseline lies just above SSP3-7.0. From the baseline, we derive a history of emissions assuming a constant 120-y lifetime, which we then use with various assumptions for the changing lifetime.

We consider the extrapolation of two climate-driven trends in N_2_O lifetime: –1.4% dec^−1^ (thick black solid line) derived here and –2.1% dec^−1^ (thick black dashed line) from ref. 9. These choices are informative since the reduction in lifetime is equivalent to a shift to two lower emission scenarios: SSP4-6.0 and SSP5-8.5, respectively. A full chemistry-climate model ECHAM6 (40) ran a standard +1%/y CO_2_ warming scenario and found that the N_2_O lifetime decreases linearly with mean warming: –20% at +3 °C. The SSP3-7.0 scenario projects warming of about 2.5 °C for our period 2020 to 2100 (41), and scaling the ref. (40) results gives an N_2_O lifetime that decreases fortuitously close to our –2.1% dec^−1^, supporting our conclusion that the current decline in N_2_O lifetime is driven by global warming.

The chemical feedback of N_2_O on its own lifetime (19) is well established with multimodel studies (table 4.5 of ref. 42) and an IPCC recommended value of –5%. More recent 3D model studies (15) found a decay time for the N_2_O mode that was smaller (108 y) than the steady-state lifetime (118 y) derived from surface emissions. Yet, the 108-y mode has an amplitude that is 1.02 times larger than the added burden (i.e., the decay of a tropospheric N_2_O perturbation does not start until it reaches the tropical middle stratosphere, about 2 y). Thus, the best current feedback factor that relates lifetime change to burden change is d(ln(N_2_O lifetime))/d(ln(N_2_O burden)) = –0.065. Combining this feedback with the –1.4% dec^−1^ lifetime trend (thin black dotted line), we find only a small additional reduction in N_2_O. While the N_2_O chemical feedback on its lifetime is a small part of the uncertainty in projecting N_2_O abundance over the 21st century, it remains an important factor in deriving the global warming potential (GWP). The 100-y GWP of N_2_O calculated using the 117 y lifetime needs to be reduced by 6.5% to account for the feedback on lifetime and an additional 4.5% to account for the reduction in CH_4_ (15).

Stratospheric chemistry and dynamics present uncertainty in projecting N_2_O that is as large as that across a different SSP emissions scenarios. The chemical feedback is much smaller and readily included with minimal uncertainty, but the trend in lifetime apparently caused by climate change and/or BDC increases is important and must be recognized in climate assessments.

## Data Availability

The data derived for this analysis, including all figures, is available at ref. 43.
